# Crossbreeding crioula and lacaune for milking productive performance and milk composition of crioula × lacaune crossbred ewes under pampa conditions

**DOI:** 10.1007/s11250-026-05208-1

**Published:** 2026-07-10

**Authors:** Eduarda Arteche Berón da Fontoura, Melina Calegaro Tamiozzo, Leonardo de Melo Menezes, Rafael Fernandes Leite, Carla Joice Härter

**Affiliations:** 1 Departamento de Zootecnia, Universidade Federal de Pelotas (UFPEL), Avenida Eliseu Maciel, s/n, Campus Capão do Leão, Capão do Leão, RS 96001970 Brasil; 2 Universidade Estadual do Rio Grande do Sul (UERGS), Unidade da Uergs em Santana do Livramento, Rua Rivadávia Corrêa, 825, Santana do Livramento, RS 97573553 Brasil; 3 Departamento de Zootecnia, Universidade Federal de São João del-Rei (UFSJ), Praça Frei Orlando, 170, São João del-Rei, MG 36307-352 Brasil

**Keywords:** Crioula, Lacaune, Crossbreeding, Milk yield, Milk composition, Pampa

## Abstract

Crioula sheep are used in tough conditions in South America, especially in the Pampa Biome, and breeding them with dairy sheep may be a good strategy for producers to improve dairy products without losing the hardiness of the sheep. Thus, the objective of this study was to evaluate the milk production characteristics of Lacaune, Crioula, and Lacaune × Crioula (F1) ewes. Thirty-seven ewes were used: 14 Lacaune, 14 Crioulas, and 9 F1 (crossbred) ewes from the same producer. All ewes grazed on Pampa native pastures with supplementation after milking. The milk production of the ewes was monitored weekly during the lactation period. On average, milk production during the entire lactation period was highest for Lacaune ewes (1076 g/day), followed by F1 (932 g/day) and Crioula (358 g/day) (*P* < 0.01). Milk from F1 ewes had intermediate concentrations of fat, protein, salts, and defatted dry extract compared to those found in the milk of Criola and Lacaune ewes (*P* < 0.01). Up to the 14th week of lactation, F1 ewes had milk yields similar to those of Lacaune ewes (*P* < 0.01). The milk yield and composition of F1 ewes in the Pampa region highlight their strong potential for commercial dairy production, particularly due to the higher total solids content, which may enhance cheese yield and processing efficiency, ultimately contributing to greater profitability for sheep milk production systems.

## Introduction

Dairy sheep farming has been expanding significantly in Brazil, and the most commonly used breed is Lacaune. Despite this expansion, the dairy sheep sector in Brazil is still in an early stage of development, characterized by small-scale production, limited formal organization, and the absence of standardized national data on milk yield, herd size, and market structure, which highlights both existing challenges and opportunities for growth. To improve the productivity of dairy sheep production systems, producers in the Brazilian Pampa region have crossbred Lacaune with Crioula.

The Lacaune breed originates from France and is notable for its productivity and extended lactation periods (Barillet et al. 2001). Crioula sheep are native to southern Brazil. This breed is suitable for wool production, which is used for industrial and artisanal purposes (Cavalcanti et al. 2017). It is a rustic breed well adapted to adverse climatic, vegetational, and soil conditions. Precocity is a strong point of the breed, with ewes and rams reaching maturity at seven and four months, respectively. The vigor of the lambs is high, owing to the maternal ability of the Crioulas (Arco, 2024). The Crioula breed is also notable for its resistance to endoparasites (Bricarello et al. 2004).

Crossbreeding native sheep adapted to local conditions with specialized breeds is a genetic improvement strategy, as the resulting crossbreed allows for increased production and usually results in lambs with greater weight gain at weaning (Černá et al. 2023; Jiménez et al. 2020; Peeters et al. 1992). The F1 ewes can achieve high levels of milk production, even surpassing their maternal breeds (Černá et al. 2023). The majority studies reporting Brazilian or Argentinian Crioula sheep were concerning about wood production (Cavalcanti et al. 2017; Peña et al. 2016). However, to our knowledge, the milk production in Crioula sheep as well as Crioula’s crossbreeding has not yet been reported in the literature. Therefore, it is necessary to better understand whether Crioula breed can be incorporated into dairy production systems. Thus, this study aimed to evaluate the milk production and chemical composition of milk from Lacaune, Crioula, and F1 crossbred ewes. Our hypothesis is that crossbreeding Crioula with Lacaune sheep can improve milk composition, milk yield and lactation persistence.

## Materials and methods

### Study site, animals, and management

This study was conducted at Cabanha do Cerro da Vigia, located in Santana do Livramento, Rio Grande do Sul, Brazil. The climate classification is Cfa (Köppen and Geiger 1928), which is characterized as a subtropical climate with hot summers. The average temperature throughout the year is 18.1 °C and the average annual rainfall is 1532 mm. Humane animal care and handling procedures were conducted in accordance with the guidelines recommended by the Committee on Ethics in Animal Use at the Center of Rural Sciences of the Universidade Federal de Pelotas (#141/2021).

The study used 37 ewes, 14 Crioula breed, 14 of Lacaune, and 9 F1. The ewes were between the 1st and 3rd order of lactation. All the ewes used in the experiment were born and raised on the property and had single lambs born to them. The ewes grazed on Pampa native pasture during the day and spent the night in a barn with ad libitum access to water and minerals. Daily, ewes receive 1% of their BW as commercial concentrate with 18% crude protein (CP) after milking. On rainy days, the ewes were kept inside the shed all day and were provided concentrate, corn silage, minerals, and water.

### Milking procedures and milk analysis

The ewes were milked daily, at 05:00 AM and 04:00 PM using a milking machine (Implemis, model Ord300, Santa Rosa, RS, Brazil). Measurements and collections of milk started after weaning, which occurred at 45 days of life (approximately 6.4 weeks after parturition), and the days in milk (DIM) were recorded. Milk production was measured daily using an adapted Milk Meter for sheep (Agropeperi, Itapiranga, SC, Brazil) coupled to a milking machine tube. Milk production measurements and milk samples (approximately 30 ml) were collected once a week from the 7th week after parturition to the 21st week, which was near the end of lactation for these ewes. Milk sampling was performed only during the morning milking and immediately analyzed for its composition using a Master Mini milk analyzer (AKSO, São Leopoldo, RS, Brazil). The variables measured were fat, protein, lactose, salts (mainly potassium, calcium, phosphates, citrates, chlorides, sulfates, and magnesium), defatted dry extract (DDE), density, and freezing point (FP). During the experimental period, the ewes were weighed every 30 d.

### Lamb management and performance

Thirty-seven lambs (20 males and 17 females) were weighed at birth and weaning, and their average daily gain (ADG) was calculated. The lambs remained with their mothers in the pasture during the day, and during the night, the lambs were separated from their mothers. From 10 days of life, a concentrate with 18% CP was offered to the lambs, composed of a mixture of soybean meal, ground corn, and powdered milk. The lambs were weaned at 45 days of age.

### Statistical analysis

Milk production and composition were analyzed considering the fixed effects of ewes’ genotypes (Crioula, Lacaune, and F1) and weeks of lactation as repeated measures over time. Analysis of variance was performed using the MIXED procedure in SAS. The covariance matrix was tested to determine the best fit to the data using the AICC as a selection criterion, and the variance component structure (VC) was found to be the best. The means were compared using Fisher’s test (DIFF option of the LSMEANS statement), and differences were considered statistically significant at < 5% probability. Lactation order was tested as a covariate and remained in the model when it was significant (*P* < 0.10).

The average weight of ewes, lambs’ birth and weaning weights, and average daily gain (ADG) were analyzed considering the genotype effect as a mixed model. The lamb weights were also tested for the effect of lamb sex. The means were compared using Fisher’s test (DIFF option of the LSMEANS statement), and differences were considered statistically significant at < 5% probability.

## Results

Lacaune ewes produced the highest milk yield, F1 ewes had an intermediate yield, and Crioula ewes produced the lowest yield (*P* < 0.01; Table 1). Nevertheless, F1 ewes produced approximately 87% of the total milk yield of Lacaune ewes and 160% more milk than Crioula ewes. In addition, DIM of F1 ewes was similar to Lacaune ewes (*P* < 0.01; Table 1). Milk from Crioula ewes had the highest concentrations of fat, protein, salts, and DDE, whereas the milk from F1 ewes had intermediate concentrations, and the lowest concentrations were found in milk from Lacaune ewes (Table 1; *P* < 0.01). The milk FP was the lowest in Crioula ewes, intermediate in F1 ewes, and the highest in Lacaune ewes (*P* < 0.01; Table 1). The milk lactose content of Crioula ewes was greater than that of F1 and Lacaune ewes (*P* < 0.01; Table 1). The milk densities of the Crioula and F1 ewes were similar but greater than that of the Lacaune ewes (*P* < 0.01; Table 1).

**Table 1:** Average milk yield, composition, and physical characteristics of Crioula, Crioula × Lacaune (F1), and Lacaune ewes

Variable	Genotype									*P*-value	
	Crioula	SEM¹	SD²	F1	SEM	SD	Lacaune	SEM	SD		
Milk yield (g/day)	358^c^	40.3	223	932^b^	25.7	446	1076^a^	20.7	482		< 0.01
DIM³	80.8^b^	5.54	27.2	133^a^	6.91	12.6	137^a^	5.54	16.9		< 0.01
Fat (%)	7.28^a^	0.123	1.26	6.60^b^	0.097	1.10	6.38^c^	0.077	1.11		< 0.01
Protein (%)	4.47^a^	0.021	0.183	4.38^b^	0.017	0.184	4.32^c^	0.013	0.193		< 0.01
Lactose (%)	6.59^a^	0.029	0.232	6.47^b^	0.023	0.236	6.42^b^	0.018	0.256		< 0.01
Salts (%)	0.978^a^	0.004	0.033	0.959^b^	0.003	0.037	0.946^c^	0.002	0.032		< 0.01
Defatted dry extract (%)	12.1^a^	0.045	0.402	11.9^b^	0.036	0.390	11.7^c^	0.028	0.389		< 0.01
Density (kg/m³)	1038.68^a^	0.180	1.45	1038.41^a^	0.142	1.66	1038.01^b^	0.111	1.47		< 0.01
Freezing point (º C)^4^	-0.92^a^	0.0044	0.038	-0.89^b^	0.0038	0.035	-0.87^c^	0,0029	0.039		< 0.01

¹SEM=Standard error of the Mean, ²SD= Standard deviation; ³ DIM=Days in milk; ^4^Number of lactation was a significant covariate in this model (*P* < 0.05)

^a−c^ Means with different superscript letters are significantly different by Fisher’s test

The birth weight, weaning weight, and ADG of lambs were similar among the breeds (Table 2). There was only an effect of sex on birth weight (*P* = 0.04), in which males were heavier than females, weighing an average of 4.2 kg (± 0.106) and females an average of 3.8 kg (± 0.130).

**Table 2:** Body weights of ewes, lambs born from Crioula, Crioula × Lacaune (F1), and Lacaune ewes, and their average daily gain (ADG)

Variable	Genotype									*P*-value	
	Crioula	SEM¹	SD²	F1	SEM	SD	Lacaune	SEM	SD		
Ewe body weight³ (kg)	44.3^c^	1.18	5.36	47.7^b^	1.04	6.15	52.5^a^	0.846	7.50		< 0.01
Lambs birth weight* (kg)	3.95	0.133	0.377	3.92	0.173	0.471	4.05	0.130	0.544		0.78
Lambs weaning weight (kg)	12.3	0.432	1.54	11.6	0.562	0.829	11.2	0.422	1.59		0.23
ADG (g)	187	8.63	0.029	172	11.2	0.018	159	8.44	0.032		0.12

¹SEM=Standard error of the Mean; ²SD= Standard deviation; ³Sex had a significant effect on the lambs’ birth weight (*P* = 0.04); males were born heavier (4.18 ± 0.106) than females (3.79 ± 0.130)

**Fig. 1:**
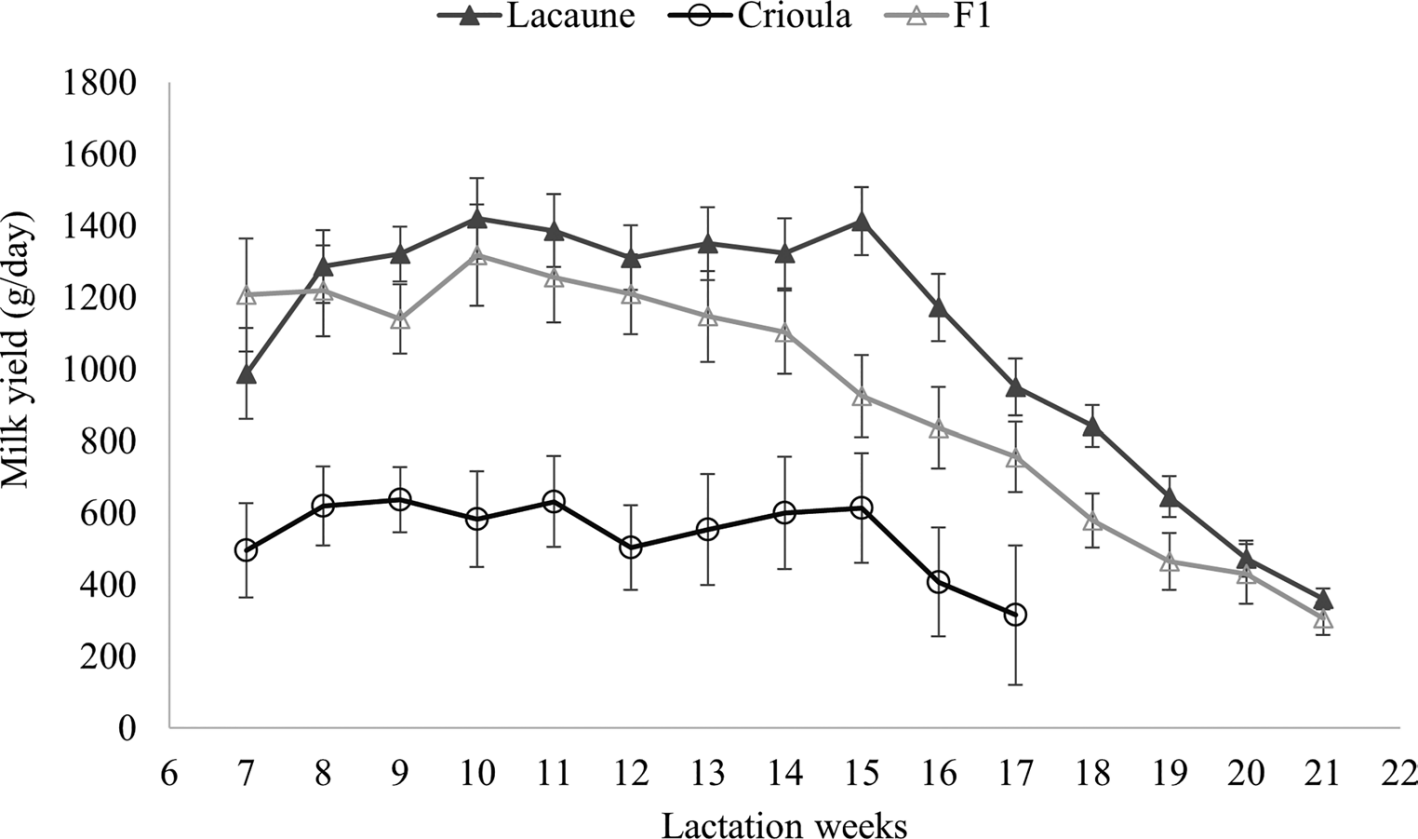
Average daily milk yield of Crioula (○), Crioula × Lacaune (F1; Δ), and Lacaune (▲) ewes from 7 to 21 weeks of lactation

Throughout the entire lactation period, Crioula ewes produced less milk than Lacaune and F1 ewes (*P* < 0.01; Fig. 1). Up to the 14th week of lactation, F1 ewes had milk yields similar to those of Lacaune ewes (*P* < 0.01). As expected, milk production declined towards the end of lactation (*P* < 0.01; Fig. 1). For Lacaune and Crioula ewes, this decrease started after the 16th week. However, F1 ewes experienced an earlier drop, with a significant reduction observed by the 15th week (Fig. 1).

**Fig. 2:**
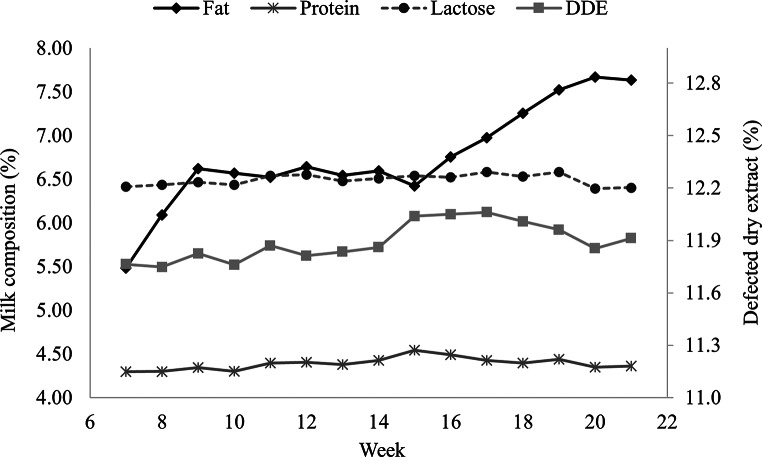
Average protein (⁎), fat (♦), lactose (●), and defatted dry extract (DDE; ■) composition of ewes’ milk (Crioula, Crioula × Lacaune (F1), and Lacaune) from 7 to 21 weeks of lactation

During lactation, the ewes maintained stable protein levels in their milk, ranging from 4.1% to 4.5% (*P* < 0.01; Fig. 2). The fat content gradually increased over the weeks of lactation, with the highest levels observed after the 15th week of lactation (*P* < 0.01; Fig. 2). The concentration of DDE was higher after the 15th week of lactation (*P* < 0.05; Fig. 2). Conversely, the FP decreased linearly with lactation advance from − 0.86º C to -0.91º C (*P* < 0.01; Fig. 2).

## Discussion

Crossbreeding between Lacaune and Crioula sheep is a viable alternative for dairy production, as it allows the combination of the productive characteristics of Crioula sheep in the Pampa region with the high milk yield of the Lacaune breed. This is supported by the fact that Lacaune ewes, as expected, showed higher milk production due to their dairy aptitude. However, F1 ewes had milk yields very close to those of Lacaune sheep, especially during the first two-thirds of the lactation period. Robles Jimenez et al. (2020), in research conducted under conditions comparable to those of the present study and involving crossbreeding Lacaune ewes with Manchega sheep, reported that although crossbred ewes exhibited greater lactation persistence, pure Lacaune ewes had higher milk yields. In addition, in the present study, the F1s presented intermediate productive performance, with production equivalent to 87% of that of Lacaune ewes, but 160% higher than that of Crioulas, and presented DIM similar to that of Lacaune ewes, which is relevant for increasing total milk production. Previous studies have reinforced the importance of lactation persistence as a determinant of final yield, since a higher DIM is directly associated with accumulated production (Macciotta et al. 2011; Elvira et al. 2013). The similar milk yield of F1 and Lacaune ewes up to the 14th week of lactation is economically relevant, as this period accounts for a substantial proportion of total milk production in commercial dairy sheep systems. Moreover, the higher total solids content in F1 milk enhances its industrial value, particularly for cheese production, potentially offsetting the sharper decline in yield after the 14th week by increasing processing efficiency per unit of milk. Therefore, although Crioula showed lower milk production and shorter persistence, crossbreeding did not impair F1 milk yield, which benefited from the productive gains associated with Lacaune genetics.

In terms of composition, the high concentration of solids in the milk from Crioula sheep is in accordance with the findings of Larrosa and Kremer (1990), whereas Lacaune sheep produced greater volumes with lower solid concentrations, a pattern already described for this breed (Barillet et al. 2001; Brito et al. 2006). The observed milk composition of F1 ewes may ensure both quality and volume, a result consistent with the heterosis effect described in dairy sheep crossbreeding (Ferreira et al. 2011; Jiménez et al. 2020). This balance between production and composition may represent a potential advantage in artisanal cheese production systems, where solid content is known to be crucial for industrial yields (Fava et al. 2014), though the direct impact on cheese yield was not assessed in the present study. In this context, the intermediate milk composition observed in F1 ewes has important practical implications for small-scale dairy systems. Because F1 milk presents balanced levels of fat, protein, and total solids, it tends to provide a raw material with more stable behavior during processing and more predictable technological performance, as reported for sheep milk destined for cheese production (Park et al. 2007; Ferreira et al. 2011). For artisanal producers, this composition is advantageous because it supports a consistent transformation of milk into value-added products, reducing variability during coagulation and cheese yield (Penna 2011). In practical terms, the intermediate profile of F1 milk could contribute to greater uniformity of the final product and could enhance the economic attractiveness of these animals for farms engaged in small-scale dairy commercialization.

Lactose content is positively related to milk production, as the amount of milk produced in the mammary gland depends on the capacity for lactose synthesis, which is a limiting factor for production (Alais 1985). In this sense, higher lactose levels can be attributed to greater milk production. However, this pattern was not observed in the Crioula sheep evaluated, which, despite having relatively high lactose levels, produced less milk than other genotypes. These results suggest that other factors may influence milk production in Crioula sheep, possibly related to evolutionary adaptations to more extensive production systems, where metabolic efficiency and the ability to maintain production under lower nutritional input are favored over higher milk volumes, as well as the efficiency in synthesizing other milk components (such as fat and protein). Based on this, future studies should explore whether the observed lactose levels are directly associated with udder health or whether other physiological and environmental variables influence milk production and composition, especially in native or adapted breeds.

Lactation progression is one of the main factors affecting milk composition (Albenzio et al. 2004; Sevi et al. 2004). Higher fat content is expected in the first weeks, gradually declining until the establishment of the peak and persistence phases of lactation, with a new increase in the final lactation period (Fontoura et al. 2020). Although milk composition during the first weeks of lactation was not evaluated in the present study, the increase in fat content observed in the last weeks of lactation aligns with other studies that reported a progressive increase in fat concentration as lactation advances in sheep (Bianchi et al. 2004; Albenzio et al. 2005; Oliveira 2006). In addition to the increase in fat concentrations, the increase in DDE observed in the last weeks of lactation may also be directly related to the decrease in milk production (Brito et al. 2006; Fava et al. 2014). As the DDE concentration increases, the FP value decreases (TRONCO, 1997). This explains the reduction in the FP observed as lactation progressed, especially in the last weeks, since these are directly related to DDE levels.

Regarding lamb performance, the results observed suggest that regardless of the greater milk volume produced by Lacaune and F1, the management conditions and supplementation provided to the lambs were sufficient to equalize the performance between groups. Only the effect of sex was significant, with male lambs presenting higher birth weights, which agrees with the literature on sexual dimorphism in small ruminants (Gardner et al. 2007; Robinson et al. 1977).

In general, the use of local breeds, such as Crioula, in crossbreeding programs enhances milk composition in F1 ewes compared to pure Lacaune, increasing total solids content, which is particularly relevant for cheese production according to the literature. Crossbreeding strategies have been recommended because they enhance the complementarity between breeds, resulting in greater productive efficiency and system sustainability (Barillet 2007; Carta et al. 2009). Therefore, this study indicates that the use of F1 females, resulting from crossbreeding between Crioula and Lacaune, is a promising alternative for dairy systems in southern Brazil, as it combines strong productive performance and lactation persistence with improved milk composition, particularly higher total solids. These results may contribute to enhance cheese yield and processing efficiency and increase the profitability of sheep dairy systems in the Pampa region; therefore, further studies should be conducted to evaluate such aspects.

Although the results provide consistent evidence of the productive advantages of crossbreeding under the conditions evaluated, this study was conducted with a limited number of animals and on a single farm. These factors do not compromise the internal validity of the findings but indicate that future research should be carried out with larger populations and across different production environments. Expanding the scale of evaluation would help confirm the robustness of the observed responses and support broader recommendations for crossbreeding strategies in dairy-sheep systems.

## Conclusion

The milk production, lactation persistence, and milk composition of F1 ewes indicate that crossbreeding Crioula with Lacaune is a viable strategy for dairy systems in the Pampa region. These characteristics suggest that F1 ewes can be integrated into commercial dairy operations and may provide additional advantages for cheese production due to their enhanced milk quality.

## Data Availability

Data will be made available upon request.

## References

[CR1] Alais C (1985) Ciencia de la leche: Principios de técnica lechera, 4th edn. Editorial Reverté S.A, Barcelona

[CR2] Albenzio M, Caroprese M, Santillo A, Marino R, Taibi L, Sevi A (2004) Effects of somatic cell count and stage of lactation on the plasmin activity and cheese-making properties of ewe milk. J Dairy Sci 533:542–587. 10.3168/jds.S0022-0302(04)73194-X 10.3168/jds.S0022-0302(04)73194-X 15202636

[CR3] Albenzio M, Caroprese M, Santillo A, Marino R, Taibi L, Sevi A (2005) Proteolytic patterns and plasmin activity in ewe’s milk as affected by somatic cell count and stage of lactation. J Dairy Res 86:92–72 10.1017/s0022029904000676 15747735

[CR4] ARCO. Associação Brasileira de Criadores de Ovinos (2024) Crioula. Available at: http://www.arcoovinos.com.br/index.php/mn-srgo/mn-padroesraciais?id=44. Accessed 14 Oct 2024

[CR5] Barillet F (2007) Genetic improvement for dairy production in sheep and goats. Small Rumin Res 60:75–70. 10.1016/j.smallrumres.2007.01.004

[CR6] Barillet F, Marie C, Jacquin M, Lagriffoul G, Astruc JM (2001) The French Lacaune dairy sheep breed: use in France and abroad in the last 40 years. Livest Prod Sci 17:29–71. 10.1016/S0301-6226(01)00237-8

[CR31] Bianchi L, Casoli C, Pauselli M, Budelli E, Caroli A, Bolla A, Duranti E (2004) Effect of somatic cell count and lactation stage on sheep milk quality. Ital J Anim Sci 3(2):147–156. 10.4081/ijas.2004.147

[CR7] Bricarello PA, Gennari SM, Oliveira-Sequeira TCG, Vaz CMSL, Gonçalves I, Echevarria FAM (2004) Worm burden and immunological responses in Corriedale and Crioula Lanada sheep following natural infection with Haemonchus contortus. Small Rumin Res 75:83–51. 10.1016/S0921-4488(03)00188-3

[CR8] Brito MA, González FHD, Ribeiro LAO, Campos R, Lacerda LA (2006) Composição do sangue e do leite em ovinos leiteiros do sul do Brasil: variações na gestação e na lactação. Ciênc Rural 942:948–936. 10.1590/S0103-84782006000300033

[CR9] Carta A, Casu S, Salaris S (2009) Invited review: Current state of genetic improvement in dairy sheep. J Dairy Sci 5814:5833–5892. 10.3168/jds.2009-2479 10.3168/jds.2009-2479 19923587

[CR10] Cavalcanti L, Moraes J, Faria D, McManus C, Nepomuceno A, Souza C, Caetano A, Paiva S (2017) Genetic characterization of coat color genes in Brazilian Crioula sheep from a conservation nucleus. Pesquisa Agropecuaria Brasileira 52:615–622. 10.1590/s0100-204x2017000800007

[CR11] Černá M, Margetín M, Veselá Z, Milerski M (2023) Effects of crossbreeding on milk production of sheep. Czech J Anim Sci. 10.17221/39/2023-cjas

[CR30] Elvira L, Hernandez F, Cuesta P, Cano S, Gonzalez-Martin JV, Astiz S (2013) Factors affecting the lactation curves of intensively managed sheep based on a clustering approach. J Dairy Res 80(4):439–447. Epub 2013 Sep 4. PMID: 24000902. 10.1017/S0022029913000381 10.1017/S0022029913000381 24000902

[CR12] Fava LW, Fischer V, Zanela MB, Quadros SAF (2014) Evaluation of physicochemical characteristics of fresh, refrigerated and frozen Lacaune ewes’ milk. Arq Bras Med Vet Zootec 1924(6):1930. 10.1590/1678-7675

[CR13] Ferreira MIC, Borges I, Macedo Junior GL, Rodriguez NM, Penna CFAM, Souza MR, Gomes MGT, Souza FA, Cavalcanti LF (2011) Produção e composição do leite de ovelhas Santa Inês e mestiças Lacaune e Santa Inês e desenvolvimento de seus cordeiros. Arq Bras Med Vet Zootec 63(2):530–533. 10.1590/S0102-09352011000200040

[CR14] Fontoura EAB, Moraes CM, Nörnberg JL, Fischer V (2020) Características da lactação de ovelhas Texel criadas extensivamente. Braz J Dev 1586:1597–1596. 10.34117/bjdv6n1-109

[CR15] Gardner DS, Buttery PJ, Daniel Z, Symonds ME (2007) Factors affecting birth weight in sheep: maternal environment. Reproduction 297:307–133. 10.1530/REP-06-0042 10.1530/REP-06-0042 PMC1994721 17244755

[CR16] Jiménez L, Hernandez J, Palacios C, Abecia J, Naranjo A, Avalos J, Gonzalez-Ronquillo M (2020) Milk Production of Lacaune Sheep with Different Degrees of Crossing with Manchega Sheep in a Commercial Flock in Spain. Animals: Open Access J MDPI 10. 10.3390/ani10030520 10.3390/ani10030520 PMC7143488 32244918

[CR32] Köppen W, Geiger R (1928) Klimate der Erde, Gotha. Wall-map 150cmx200cm

[CR17] Larroza JR, Kremer R (1990) Leche ovina y caprina – Una nueva alternativa agroindustrial. Editorial Hemisferio Sur, Montevideo, p 172

[CR18] Macciotta NPP, Dimauro C, Rassu SPG, Steri R, Pulina G (2011) The mathematical description of lactation curves in dairy cattle. Ital J Anim Sci 10(4). 10.4081/ijas.2011.e51

[CR19] Oliveira VLM (2006) Aspectos do leite e mastite em ovinos da raça Santa Inês em Sergipe. Master’s Dissertation, Universidade Federal de Sergipe. Available at: http://www.dominiopublico.gov.br/download/texto/cp010533.pdf. Accessed Dec 2021

[CR20] Park YW, Juárez M, Ramos M, Haenlein GFW (2007) Physico-chemical characteristics of goat and sheep milk. Small Rumin Res 68:88–113. 10.1016/j.smallrumres.2006.09.013

[CR21] Peeters R, Buys N, Vanmontfort D, Vandepitte W 91992) Milk yield and milk composition of Flemish Milksheep, Suffolk and Texel ewes and their crossbreds. Small Rumin Res 279:288-7. 10.1016/0921-4488(92)90162-W

[CR22] Peña S, Sacchero D, Mauriño J, López G, Abbiati N, Género E, Martínez R (2016) Caracterización de la lana de ovejas Criollas argentinas en cuatro ambientes diferentes. Arch De Zootecnia 65:13–19. 10.21071/az.v65i249.436

[CR23] Penna CFAM (2011) Produção e parâmetros de qualidade de leite e queijos de ovelhas Lacaune, Santa Inês e suas mestiças submetidas a dietas elaboradas com soja ou linhaça. PhD Thesis, Federal University of Minas Gerais

[CR25] Robinson JJ, McDonald I, Fraser C, Crofts RMJ (1977) Studies on reproduction in prolific ewes: I. Growth of the products of conception. J Agric Sci 539:552–558. 10.1017/S0021859600037229

[CR24] Robles Jimenez LE, Angeles Hernandez JC, Palacios C, Abecia JA, Naranjo A, Osorio Avalos J, Gonzalez-Ronquillo M (2020) Milk Production of Lacaune Sheep with Different Degrees of Crossing with Manchega Sheep in a Commercial Flock in Spain. Anim (Basel) 10(3):520. 10.3390/ani10030520 10.3390/ani10030520 PMC7143488 32244918

[CR26] Sevi A, Albenzio M, Taibi L, Muscio A, Annicchiarico G (2004) Effects of lambing season and stage of lactation on ewe milk quality. Small Rumin Res 251:259–251. 10.1016/S0921-4488(03)00196-2

[CR27] Tronco VM (1997) Manual para inspeção da qualidade do leite, 4th edn. UFSM, Santa Maria, p 206

